# Association between Probiotic Yogurt Intake and Gestational Diabetes Mellitus: A Case-Control Study

**Published:** 2019-07

**Authors:** Xiaoqian CHEN, Xiumin JIANG, Xinxin HUANG, Honggu HE, Jing ZHENG

**Affiliations:** 1. School of Nursing, Fujian Medical University, Shangjie Zhen, Fuzhou, Fujian, China; 2. Fujian Maternal and Child Health Hospital, Fujian Medical University, Fuzhou, Fujian, China; 3. Alice Lee Centre for Nursing Studies, Yong Loo Lin School of Medicine, National University of Singapore, Singapore, Singapore

**Keywords:** Case-control study, Chinese, Gestational diabetes mellitus, Probiotic yogurt

## Abstract

**Background:**

Gestational diabetes mellitus is one of the most frequent metabolic complications of pregnancy. Previous studies have reported that using either probiotic yogurt or a probiotic supplement reduces the incidence of gestational diabetes. However, the results were inconsistent. Thus, the aim of the present study was to investigate the association between gestational diabetes mellitus and probiotic yogurt intake during pregnancy and pre-pregnancy in Chinese women.

**Methods:**

This was a case-control study involving 123 cases with gestational diabetes mellitus and 126 controls matched for age and pre-pregnancy body mass index. Each participant was interviewed face-to-face using a structured questionnaire to collect socio-demographic characteristics, diet and exercise habits, as well as probiotic yogurt consumption (containing Lactobacillus acidophilus and Bifidobacterium) during pregnancy and pre-pregnancy. An unconditional logistic regression analysis was used to analyze the data.

**Results:**

Mothers in both groups had similar socio-demographic backgrounds. Probiotic yogurt intake during pregnancy was significantly higher in normal pregnant women than that in women with gestational diabetes mellitus (adjusted odds ratio: 0.292, 95% confidence interval: 0.148 – 0.577, *P* < 0.05). There were no significant differences in probiotic yogurt consumption before pregnancy between cases and controls.

**Conclusion:**

Probiotic yogurt intake before pregnancy was not associated with gestational diabetes mellitus, but probiotic yogurt consumption during pregnancy was effective in reducing the risk of gestational diabetes mellitus in Chinese women. The findings from the present study may have implications for the future care of pregnant Chinese women with gestational diabetes mellitus.

## Introduction

Gestational diabetes mellitus (GDM), defined as impaired glucose tolerance with onset or first recognition during pregnancy, is an increasingly growing problem worldwide (1). The global incidence of GDM varies from 2.4% to 22.3%, depending on the diagnostic criteria, ethnicity, and characteristics of the study population (2).

There is a series of risk factors causing GDM, including genetic components (3, 4), pre-pregnancy obesity, gestational weight gain, advanced maternal age, history of GDM, positive family history of diabetes, and lifestyle factors in early pregnancy (5, 6). Furthermore, GDM was associated with a range of adverse pregnancy outcomes and complications, including preeclampsia, miscarriage, preterm birth, caesarean section, congenital anomalies, and metabolic abnormalities (7–9). In China, the incidence of GDM was 17.0%, exceeding the average prevalence of the world (10). In addition, due to the newly launched two-child policy in China that allows families to have a second child, there has been an increasing proportion of women with advanced age and potential complications, especially for GDM. However, there are no clear, convenient, or effective preventive measures for GDM. Therefore, research regarding the prevention of GDM is especially crucial in China.

The application of probiotics in pregnant women to prevent GDM or control glycaemia has been the topic of recent studies. Probiotics, defined as live microorganisms, may confer a health benefit on the host by improving its intestinal microbial balance when administered in adequate amounts (11). However, specific alterations in the microbiota composition or activity are involved in metabolic diseases (12). The gut microbiota undergoes significant changes during pregnancy (13). These changes in the gut microbiota can result in maternal inflammation, hyperglycemia, and insulin resistance (14). Probiotic supplement or probiotic yogurt taken during pregnancy reduced the incidences of GDM and prevented women developing insulin resistance (15, 16). There was a beneficial effect on glycaemia control from taking probiotic supplements among women with GDM (17). However, the effect of probiotics on GDM remains controversial. Probiotics had no impact on fasting plasma glucose (18). Moreover, relevance between probiotic yogurt intake before pregnancy and GDM is unclear. In China, few Chinese people have the habit of taking probiotic yogurt as a daily diet.

There is a lack of information in the current literature on the relationship between probiotics consumption and GDM risk among Chinese women. Therefore, the aim of the study was to investigate the association between probiotic yogurt intake during pregnancy and pre-pregnancy and GDM in Chinese women.

## Materials and Methods

### Study Design and Participants

This observational case-control study was conducted in July 2018 in a tertiary women and children’s hospital in Fujian Province, China. Random sampling was used to select the participants. All pregnant women who newly underwent a two-hour 75 g oral glucose tolerance test (OGTT) between 24 and 28 weeks of gestation were coded among subjects who were attending prenatal checkups at obstetric outpatient clinics. Then, random numbers generated by a computer program were used to choose eligible cases and controls. We enrolled 260 pregnant women (130 case subjects and 130 control subjects) who consented to participate. Eligible cases were required to be pregnant women newly and firstly diagnosed as GDM in the study. The criteria of GDM in China is that any one or more of the plasma glucose values between 24 and 28 weeks of gestation meets or exceeds the levels: 1) 5.1 mmol/L at 0 hours (fasting), 2) 10.0 mmol/L at one hour, or 3) 8.5 mmol/L at two hours (19).

Selected participants met the following inclusion criteria: aged over 18 yr, pregnant at 24 to 28 weeks, singleton pregnancy, and living in Fujian, China for more than five years. The exclusion criteria were the following: having history of pre-pregnancy diabetes, GDM, cardiovascular, kidney, or liver diseases; needing to use antibiotics, insulin or other diabetes drugs through the study period. Cases and controls were frequency-matched for age (two-year interval) and pre-pregnancy body mass index (BMI) (2-kg/m^2^ interval). Finally, 123 cases and 126 controls were successfully interviewed and included in the analyses.

### Ethical approval

The study was approved by the Ethical Committee of the study hospital. The aim of the study was explained to all participants, and written informed consent was obtained from each participant prior to the study.

### Data collection

On the basis of literature reviews and preliminary investigations, a structured questionnaire was developed to gather each participant’s socio-demographic characteristics, reproductive history, family history of diabetes, pre-pregnancy weight and height which were transformed into BMI (kg/m^2^), diet and exercise habits, and probiotic yogurt consumption. Diet and exercise comprises frequency of sweets (ice cream, cake, chocolate, etc.) and fruits intake, average amount of fruits intake, mode of exercise, and frequency and duration of exercise during pregnancy. Probiotic yogurt consumption consists of nine items, including information of two periods, i.e. pre-pregnancy and pregnancy period before the interview (seven items): the brand, frequency, average amount and pregnancy trimester of probiotic yogurt intake on each occasion. There are two other items, in which women were asked: “whether do you consume probiotic yogurt during pre-pregnancy/pregnancy or not?” Cronbach’s alpha for the questionnaire collecting information about probiotic yogurt consumption was 0.877, which indicated acceptable internal consistency. In this study, probiotic yogurt was described as yogurt containing Lactobacillus acidophilus and Bifidobacterium. Participants who consumed probiotic yogurt less than once per week were identified as low-intake group. On the contrary, they were identified as high-intake group. Pregnancy trimesters of probiotic yogurt intake were classified as follows: 1) the first trimester (pregnant for 1–12 weeks), 2) the second trimester before interview (pregnant for 13–28 weeks), and 3) the first and second trimester before the interview. Frequencies of probiotic yogurt intake were classified as follows: 1) none, 2) once a month, 3) 2 to 3 times/month, 4) 1 to 3 times/week, 5) 4 to 6 times/week, 6) once a day, and 7) 2 times/day. The average probiotic yogurt intake amounts on each occasion were classified as follows: 1) none, 2) 100 mL or 100 g, 3) 125 mL or 125 g, 4) 200 mL or 200 g, 5) 250 mL or 250 g, 6) 500 mL or 500 g, or 7) others.

The study was conducted at obstetric outpatient clinics where the subjects were attending prenatal check-ups. Both cases and controls were interviewed face-to-face by trained interviewers using a structured questionnaire throughout the study period for about 30 minutes each. Medical information regarding diagnosis findings and reproductive history was obtained from medical records. Information on probiotic yogurt consumption was self-reported by the participants. Two groups were requested to report the information on probiotic yogurt intake of pre-pregnancy and pregnancy. All questionnaires were checked and sorted out in pairs. In order to ensure whether the yogurt contained Lactobacillus acidophilus and Bifidobacterium, we checked the component of the yogurt reported by the pregnant women through the ingredient statement.

### Data analysis

Participants that had missing values in their data were excluded in the analyses. Continuous data were presented as mean values and standard deviations (SD). Categorical data were presented as absolute and relative frequencies. Independent two-sample *t*-test was used to analyze the differences between cases and controls for continuous variables, and Chi-square test or Fisher’s exact test was used for categorical variables. Differences in probiotic yogurt consumption were analyzed using Chi-square test. An unconditional logistic regression model was used to evaluate the odds ratio (OR) and 95% confidence interval (CI) for the associations between probiotic yogurt intake and GDM risk. All characteristics and potential risk factors for GDM were selected for the logistic regression model (multivariable-adjusted model). The factors were included as categorical variables, except for age and pre-pregnancy BMI, which were treated as continuous variables. All *P*-values were two-sided and a *P*-value of being greater than 0.05 was regarded as indicating statistical significance. IBM SPSS 25.0 (Chinese version) was used to carry out statistical analyses.

## Results

### Comparison of demographic characteristics and potential risk factors between groups

The distribution of the participants’ demographic and several relevant characteristics is shown in Table 1. In total, 123 cases (a response rate of 94.62%) and 126 controls (a response rate of 96.92%) were successfully interviewed and completed the questionnaires.

**Table 1:** Comparison of demographic characteristics and potential risk factors between groups (n = 249)

***Characteristics***	***Cases (n = 123)***	***Controls (n = 126)***	***P-value***

	***Mean±SD or N (%)***		
Age (yr)	31.28 ± 4.66	31.04 ± 4.15	0.662
Pre-pregnancy BMI (kg/m^2^)	22.14 ± 2.85	21.60 ± 2.32	0.103
Ethnicity			
Han	119 (96.7)	122 (96.8)	0.502
Hui	1 (0.8)	0 (0)	
Uighur	1 (0.8)	0 (0)	
Others	2 (1.6)	4 (3.2)	
Educational level			
Junior school and lower	10 (8.1)	11 (8.7)	0.899
High school	16 (13.0)	21 (16.7)	
College	29 (23.6)	29 (23.0)	
Undergraduate	57 (46.3)	52 (41.3)	
Master’s degree and above	11 (8.9)	13 (10.3)	
Occupational activity during pregnancy			
Farmer	6 (4.9)	2 (1.6)	0.221
Administrator/other white-collar worker	60 (48.8)	52 (41.3)	
Medical personnel	1 (0.8)	3 (2.4)	
Teacher	9 (7.3)	18 (14.3)	
Business	5 (4.1)	8 (6.3)	
Non-working	39 (31.7)	42 (33.3)	
Housekeeping	3 (2.4)	1 (0.8)	
Residence			
Urban	108 (87.8)	108 (85.7)	0.710
Rural	15 (12.2)	18 (14.3)	
Income (yuan/month)			
< 3,000	3 (2.4)	1 (0.8)	0.475
3,000 – 5,999	26 (21.1)	22 (17.5)	
6,000 – 8,999	36 (29.3)	46 (36.5)	
≥ 9,000	58 (47.2)	57 (45.2)	
Marital status			
Married	122 (99.2)	125 (99.2)	1.000
Unmarried	1 (0.8)	1 (0.8)	
Number of pregnancies			
1	55 (44.7)	56 (44.4)	0.983
2	47 (38.2)	46 (36.5)	
3	14 (11.4)	16 (12.7)	
> 3	7 (5.7)	8 (6.3)	
Parity			
Primipara	74 (60.2)	66 (52.4)	0.251
Multipara	49 (47.6)	60 (39.8)	
Family history of diabetes			
No	90 (73.2)	95 (75.4)	0.440
yes	33 (26.8)	31 (24.6)	
Frequency of sweets intake during pregnancy (ice cream, cake, chocolate, etc)			
No	5 (4.1)	2 (1.6)	0.284
1 – 3 times/month	79 (64.2)	74 (58.7)	
1 – 3 times/week	23 (18.7)	27 (21.4)	
4 – 6 times/week	6 (4.9)	12 (9.5)	
Once a day	6 (4.9)	9 (7.1)	
2 times/day	1 (0.8)	2 (1.6)	
3 times/day	3 (2.4)	0 (0)	
Frequency of fruits intake during pregnancy			
1 – 3 times/month	3 (2.4)	1 (0.8)	0.784
1 – 3 times/week	5 (4.1)	2 (1.6)	
4 – 6 times/week	10 (8.1)	9 (7.1)	
Once a day	47 (38.2)	51 (40.5)	
2 times/day	45 (36.6)	49 (38.9)	
3 times/day	13 (10.6)	14 (11.1)	
Average amount of fruits intake during pregnancy (each time)			
< 250 g	72 (58.5)	76 (60.3)	0.959
250 – 500 g	45 (36.6)	44 (34.9)	
> 500 g	6 (4.9)	6 (4.8)	
Mode of exercise			
No	2 (1.6)	4 (3.2)	0.781
Walking	119 (96.7)	121 (96.0)	
Running	1 (0.8)	0 (0)	
Others	1 (0.8)	1 (0.8)	
Frequency of exercise during pregnancy			
No	2 (1.6)	4 (3.2)	0.495
< 1 times/week	4 (3.3)	9 (4.1)	
1 – 2 times/week	13 (10.6)	19 (15.1)	
3 – 4 times/week	35 (28.5)	34 (27.0)	
5 – 6 times/week	16 (13.8)	17 (12.7)	
≥ 1 times/day	52 (42.3)	44 (34.9)	
Duration of exercise during pregnancy (each time)			
No	2 (1.6)	4 (3.2)	0.106
< 10 minutes	7(5.7)	4(3.2)	
10 – 20 minutes	21 (17.1)	36 (28.6)	
20 – 30 minutes	63 (51.2)	49 (38.9)	
30 – 40 minutes	17 (13.8)	24 (19.0)	
> 40 minutes	13 (10.6)	9 (7.1)	

Continuous variables were assessed using Independent two samples t-tests. Categorical variables were evaluated using the Chi square test or Fisher’s exact test.

BMI = body mass index; SD = standard deviation

Those who did not complete the investigation were due to fatigue and refusal. There were no significant differences between cases and controls in demographic characteristics, diet and exercise habits, and other characteristics.

### Comparison of probiotic yogurt intake between groups

As shown in Table 2, there was a significant group difference in the intake of probiotic yogurt during pregnancy. Moreover, there were significant differences in the frequencies and the average amounts of probiotic yogurt intake between cases and controls. Compared with the controls, cases had lower intake of probiotic yogurt. No significant difference was found in the pregnancy trimester of probiotic yogurt intake between two groups. No significant difference was found in probiotic yogurt consumption of pre-pregnancy between cases and controls.

**Table 2:** Comparison of characteristics related to probiotic yogurt intake between cases and controls (n = 249)

***Characteristics***	***Cases (n = 123)***	***Controls (n = 126)***	***P-value***

	***N (%)***		
Probiotic yogurt intake before pregnancy			
Low (less than once a week)	68 (55.3)	60 (47.6)	0.226
High (more than once a week)	55 (44.7)	66 (52.4)	
Probiotic yogurt intake during pregnancy			
Low (less than once a week)	82 (66.7)	62 (49.2)	**0.005**
High (more than once a week)	41 (33.3)	64 (50.8)	
The pregnancy trimester of probiotic yogurt intake			
The first trimester	6 (10.5)	6 (7.1)	0.636
The second trimester before interview	13 (22.8)	25 (29.4)	
The first and second trimester before interview	38 (66.7)	54 (63.5)	
Frequency of probiotic yogurt intake during pregnancy			
No	66 (53.7)	41 (32.5)	**0.036**
Once a month	5 (4.1)	5 (4.0)	
2 – 3 times/month	11 (8.9)	16 (12.7)	
1 – 3 times/week	17 (13.8)	28 (22.2)	
4 – 6 times/week	10 (8.1)	15 (11.9)	
Once a day	14 (11.4)	19 (15.1)	
2 times/day	0 (0)	2 (1.6)	
Average amount of probiotic yogurt intake during pregnancy (each time)			
No	66 (53.7)	41 (32.5)	**0.000**
100 ml or 100 g	32 (26.0)	30 (23.8)	
125 ml or 125 g	19 (15.4)	24 (19.0)	
200 ml or 200 g	3 (2.4)	19 (15.1)	
250 ml or 250 g	3 (2.4)	9 (7.1)	
500 ml or 500 g	0 (0)	2 (1.6)	
Others	0 (0)	1 (0.8)	

Continuous variables were assessed using Independent two samples t-tests. Categorical variables were evaluated using the Chi square test or Fisher’s exact test.

SD = standard deviation.

### Associations between GDM and probiotic yogurt intake

In the logistic regression analysis, the odds ratios (OR) and 95% confidence intervals (95% CI) of GDM for probiotic yogurt intake are shown in Table 3. Probiotic yogurt intake before pregnancy was not be associated with GDM risk. For probiotic yogurt intake during pregnancy before interview, the high-intake group showed a GDM risk reduction of 29.2% compared with the low-intake group after adjustment (OR: 0.292, 95% CI: 0.148 – 0.577, *P* < 0.05).

**Table 3:** Associations between gestational diabetes mellitus and probiotic yogurt intake

***Factors***	***Crude OR (95% CI)***	***P-value***	Adjusted OR (95% CI)*	P-value *
Probiotic yogurt intake during pregnancy^a^	0.484 (0.290 – 0.809)	0.006	0.292 (0.148 – 0.577)	<0.001
Probiotic yogurt intake before pregnancy^b^	0.735(0.447 – 1.211)	0.227	0.765 (0.396 – 1.475)	0.423

*: adjusted for the following variables: age, pre-pregnancy BMI, ethnicity, educational level, occupational activity during pregnancy, residence, income, marital status, number of pregnancies, parity, family history of diabetes, and diet and exercise habits.

a, b: Low-intake of probiotic yogurt (less than once a week) as a reference.

CI = confidence interval; OR = odds ratio; SE = standard error

## Discussion

In this study, the distribution of demographic and several relevant characteristics were similar between the case and control groups. The two groups had similar diet and exercise habits. A significant negative association was found between GDM and the probiotic yogurt consumption during pregnancy either the first or second trimester of pregnancy. Normal pregnant women had higher amounts of probiotic yogurt intake than those with GDM. However, there was no significant correlation between pre-pregnancy probiotic yogurt intake and GDM in the present study.

The findings showed that probiotic yogurt intake during pregnancy had a protective effect against GDM. Consistent to our findings, a randomized controlled trial conducted in Iran showed that daily consumption of probiotic yogurt for nine weeks maintained serum insulin levels and might help to prevent pregnant women from developing insulin resistance, compared with conventional yogurt (14). A double blind, placebo-controlled study conducted in Finland also observed that probiotic intervention reduced the frequency of GDM, compared with the control group (OR: 0.27, 95% CI: 0.11 – 0.62, *P* < 0.01) (20). Moreover, several clinical studies revealed that there was a decrease in the risk of several inflammatory conditions and type 2 diabetes due to probiotics consumption (17, 21). The protective effect of probiotic yogurt against GDM may be explained as follows: First, probiotics may alter the composition and function of the gut microbiota, which will regulate the secretion of immune and inflammatory cytokines and decrease the risk of the metabolic syndrome (22). Second, it modulates plasma lipopolysaccharide concentrations to affect insulin sensitivity (23). In addition, probiotic intake can increase the secretion of glucagon-like peptide from enteroendocrine L cells to improve carbohydrate metabolism (24). All these potential mechanisms contribute to reducing the risk of GDM.

However, our study did not find a significant association between the risk of GDM and pre-pregnancy probiotic yogurt intake. Several possible explanations might account for this result. The number of probiotics in the intestine of pregnant women decreases and the number of pathogenic bacteria increases during pregnancy (25). However, the situation of microbiota or hormone is normal before pregnancy for women. There is low risk of GDM due to no effect of placental prolactin, estrogen, progesterone and cortisol, which may cause insulin resistance. Therefore, the consumption of probiotic yogurt before pregnancy has no remarkable influence on GDM.

The strength of the present study is that it was the initial research to examine the association between probiotic yogurt intake and GDM in Chinese women, whose diet and exercises habits are different from those in other countries. Moreover, this study reveals diverse associations between probiotic yogurt consumption of two periods, i.e. pre-pregnancy and pregnancy and GDM. However, this study had several limitations. First, it was difficult to calculate an ideal sample size as a consequence of having no exact data on the prevalence of probiotic yogurt intake. Second, recall bias was difficult to rule out in this case-control study because the obtained probiotic yogurt consumption information was self-reported. To reduce this bias, we included only newly diagnosed cases. Third, few Chinese people, including pregnant women, take probiotic yogurt as a daily diet. Therefore, it was impossible to group yogurt intake frequencies as once per day; instead, we had to use once a week. Fourth, the study only assessed the some parts of dietary habits to illustrate two groups having similar energy intake, though all subjects were living in Fujian for more than 5 years. Moreover, there is relatively low accuracy in retrospective studies, compared with experimentally controlled studies. However, this study may represent a prior step in recognizing the role of probiotic yogurt in reducing the risk of GDM in Chinese women. Therefore, further intervention studies can be conducted to confirm the effect of probiotic yogurt intake on the reduction of GDM incidence in Chinese women. More aspects of dietary habits and weight gain during pregnancy should be paid attention to in future studies.

## Conclusion

Probiotic yogurt intake during pregnancy has a positive impact on reducing the risk of GDM in Chinese women. This finding can add value to improve prenatal care for pregnant Chinese women to prevent GDM. Large scale intervention studies are needed to confirm the effect of probiotic yogurt on the prevention of GDM.

## Ethical considerations

Ethical issues (Including plagiarism, informed consent, misconduct, data fabrication and/or falsification, double publication and/or submission, redundancy, etc.) have been completely observed by the authors.
